# Hemorrhage Occurring after the Menopause

**Published:** 1900-06

**Authors:** E. C. Davis

**Affiliations:** Atlanta, Ga., Assistant Surgeon to the Halcyon, Dr. J. B. S. Holmes’s Private Hospital; late Major and Surgeon 2d Georgia Volunteer Infantry; late Chief Surgeon of the 3d Division 4th Army Corps, U. S. A.


### HEMORRHAGE OCCURRING AFTER THE MENOPAUSE.

By E. C. DAVIS, A.B., M.D., Atlanta, Ga.,

Assistant Surgeon to the Halcyon, Dr. J. B. S. Holmes's Private Hospital; late Major and Surgeon 2d Georgia Volunteer Infantry; late Chief Surgeon of the 3d Division 4th Army Corps, U. S. A.

I have selected this subject for consideration to-day, not to evolve any profound research, but rather to draw attention to the changes which occur at the climacteric in women, and to warn many from hanging upon the convenient hook of ignorance or carelessness so many of the disturbances which occur at this period. I have been prompted more by the number of inoperable cases of cancer of the uterus which have come to Dr. Holmes's Sanatorium during my association with him than anything else to contribute the paper, that some may be prompted to make examinations more frequently of women with pelvic disturbances at this time, and diagnosticate pathological conditions early enough to offer some hope of relief through operative measures.

The menopause is a physiological condition, and should be entered upon by a woman with normal generative organs without pathological disturbances. There are, however, but few women who reach this period of life with strictly normal generative organs, but even if there be trivial deviations from a normal condition, the disturbances should also be trivial in character. Usually the so-called "flushes" (due in all probability to nervous reflexes, which produce a dilatation, and later a contraction of the superficial capillaries, leading to a sense of heat or "flush") and the few nervous symptoms should, if the change be taking place physiologically, never amount to anything serious.

In cases in which the menopause is advanced, as observed in young women requiring the removal of the tubes and ovaries for diseased conditions, the nervous symptoms are usually more severe, on account of the changes occurring more rapidly in the generative organs, due to the complete removal of these organs, instead of the slow retrograde changes which take place naturally and extend through a long period of time, usually before the complete functionating power of the ovarian tissue is lost. At the menopause, however, we must remember that the retrograde changes do not occur alone in the organs of generation, but a retrograde metamorphosis sets up in all the tissues, making them less resistant to disease, and more capable of reverting to an embryologic condition. These changes in the tissues occur in the male as well as the female—usually several years later—and have been called the "male menopause" by some writer.

It is at this time that atheromatous changes are likely to take place in the blood-vessels, malignant diseases make their appearance, and the atrophic changes of the tissues become observable. *When the menstruation has ceased, and the menopause reached, any hemorrhage from the uterus is always pathological, and should be carefully investigated to determine its cause, while the prospects of relief are favorable, even though it originates from a grave condition.*

The causes of hemorrhage at this time are:

1. *Granular endometritis*, which usually develops before the occurrence of the menopause, and is continued throughout. This condition can usually be relieved by a careful curettement under aseptic conditions.

2. *Atheroma of uterine blood-vessels*. This may be associated with the former condition, and generally occurs in quite old women, or those suffering from atheroma in other blood-vessels. Such a condition is one which offers but little prospect of relief, as the atheromatous changes have, in all probability, occurred in other blood-vessels besides those in the uterus. Here changes in diet, consisting of an abundance of well-prepared meats and fruits, may prove of benefit, with tonics and alteratives.

3. *Vasomotor relaxation*, producing a dilatation of the blood-vessels, is a possible source of hemorrhage before the complete cessation of the menstrual flow. This is often associated with granular endometritis, but when alone causing hemorrhage should give no uneasiness. An improvement in the general health by the judicious use of tonics, and directing especial attention to the nervous system, will correct this disturbance.

4. *Uterine polypus*, when present, should be readily recognized and promptly removed. If this assumes the nature of a polypoid adenoma, this can easily be removed by the sharp curette, extreme care being exercised to prevent infection, as a curetted uterus is one of the most favorable for infection from extrinsic causes.

5. *Uterine Myofibromata*. This, as a causative factor, often occurs at this period of life, and the teaching for many years has exerted a very disastrous effect upon the profession, and occasionally leads to death through neglect. At the menopause, fibromata are said often to disappear through some retrograde change; and no doubt they occasionally decrease in size, but more often do they continue to increase in size and to menace the life and health of the patient by the continuation of the hemorrhage, in addition to the possibilities of sloughing and infection of the tissues through the putrefactive changes taking place in them while breaking down, and affecting the tissues contiguous. The limits of this paper will not permit a comprehensive consideration of this part of our subject, but I wish to state as my belief that so-called fibroids, in the hands of proper operators with suitable surroundings, should be removed.

6. The last cause of hemorrhage at this time, and the one to which I wish especially to call attention, is *carcinoma uteri*. Here it is that the danger signal is held out to observing physicians, and if appreciated, years of comfort and health may be held out to the poor sufferer; but, if unnoticed and uurecognized, the poor victim is consigned to a death more horrible than can be described.

All observations go to show that, during the early history of carcinoma it is strictly local and circumscribed in its development; but later, through the lymphatics, it soon becomes disseminated throughout the contiguous tissues, reaching such a stage that no hope of relief can be offered, and all attempts in this direction are futile or tend to cast discredit upon a most laudable operation. The cervix uteri is the most frequent seat of the disease, and epithelioma is the most common form of malignant tumor. The epithelioma, as is well known, when completely removed before the invasion of the surrounding tissues, is less likely to return than any other form of malignant tumor. Malignancy rarely attacks the nulliparous uterus, or one in which there has not been one or more decided tears, and in which there is not much indurated cicatricial tissue. Hence the importance of repairing promptly these tears, and the complete removal of this weak scar tissue when formed in the cervix. Efforts to prevent conception have been given a prominent place as a causative factor of malignancy, but I have been unable to substantiate this statement, even though realizing the frowning of nature upon all unnatural acts.

The hi-tory of hemorrhage after the menopause, together with the offensive discharge—this discharge having a peculiar, almost characteristic odor—is almost pathognomonic evidence of malignancy. All such cases should be carefully examined as soon as such symptoms present themselves, and if carcinoma be detected it should be subjected to the proper treatment as early as possible. The most rational treatment is complete removal of the diseased uterus, with appendages, before an extension has taken place into the surrounding tissues, or the uterus becomes bound by adhesions produced by the malignant condition. It has been claimed by some that in the early stage of the development of carcinoma there is a non-malignant period, and if the uterus be removed at this time the recovery is more certain and complete. The probability is, that at this stage the uterus is more completely removed, and the recovery is more certain if the conditions are detected early and the removal promptly accomplished. When the uterus becomes firmly fixed and the invasion extends high up into the broad ligaments, a hysterectomy is usually useless, and but tends to hasten the fatal result. In such cases, however, a careful curettement and cauterization checks temporarily the invasion and adds to the patient's comfort.

I have endeavored in this brief paper to call attention to the frequency of carcinoma of the uterus and the insidiousness of its development at times, and to suggest more frequent investigations of hemorrhage at the menopause, in order that many lives which are yearly being sacrificed to the pernicious ravages of malignancy may be saved, and the patients so afflicted may be spared the years of suffering incident to the neglect in the prompt recognition and treatment of these cases. Either a vaginal or an abdominal hysterectomy may be performed in these cases, the plan of operation depending largely upon the choice of the operator and the experience in these various fields of surgery. If the adhesions are not very extensive, the uterus free and not much enlarged, a vaginal hysterectomy would be the choice. If the uterus is adherent or much enlarged, the abdominal route would be my preference.

I have, I trust, impressed upon you the importance of early attention to these conditions, in order that they may be benefited when it is possible.

